# Age-Related Outcomes in Patients Undergoing Coronary Angiography: In Which Subgroups Does Age Matter? Results from a Large-Scale Retrospective Registry

**DOI:** 10.3390/jcm14030928

**Published:** 2025-01-31

**Authors:** Lasse Kuhn, Tobias Schupp, Philipp Steinke, Jonas Dudda, Mohammad Abumayyaleh, Kathrin Weidner, Thomas Bertsch, Jonas Rusnak, Ibrahim Akin, Michael Behnes

**Affiliations:** 1 Department of Cardiology, Angiology, Haemostaseology and Medical Intensive Care, University Medical Centre Mannheim, Medical Faculty Mannheim, Heidelberg University, 68167 Mannheim, Germany; 2 Institute of Clinical Chemistry, Laboratory Medicine and Transfusion Medicine, Nuremberg General Hospital, Paracelsus Medical University, 90419 Nuremberg, Germany; 3 Department of Cardiology, Angiology and Pneumology, University Hospital Heidelberg, 69120 Heidelberg, Germany

**Keywords:** age, coronary angiography, coronary artery disease, heart failure, prognosis

## Abstract

**Background**: The study investigates age-related differences in the prevalence and extent of coronary artery disease (CAD), as well as long-term outcomes in a large cohort of unselected patients undergoing invasive coronary angiography (CA). The aging population, along with an increasing number of older and multi-morbid patients undergoing CA, poses challenges for healthcare systems. Despite this, studies investigating age-related differences in the long-term outcomes of unselected patients undergoing CA are limited. **Methods**: Consecutive patients undergoing invasive CA from 2016 to 2022 were included from one institution. The prognosis of patients undergoing CA stratified by pre-specified age groups (i.e., 40–<60, 60–<80 and ≥80 years) was investigated with regard to the primary endpoint of rehospitalization for heart failure (HF), as well as the risks of acute myocardial infarction (AMI) and coronary revascularization at 36 months. **Results**: From 2016 to 2022, 7520 patients undergoing CA were included with a median age of 70 years (mean: 69 years). The prevalence of CAD (61.9% vs. 71.8% vs. 77.3%; *p* = 0.001), as well as the prevalence of three-vessel CAD (21.0% vs. 31.5% vs. 36.1%) increased with age. At 36 months, patients ≥ 80 years of age had the highest rates of rehospitalization for HF, followed by patients 60–<80 years and patients 40–<60 years (28.4% vs. 23.2% vs. 14.0%; *p* = 0.001). Consequently, compared to younger patients (i.e., 40–<60 years of age), those ≥80 years of age exhibited the highest risk of HF-related rehospitalization (≥ 80 years: HR = 2.205; 95% CI 1.884–2.579; *p* = 0.001), followed by those 60–< 80 years (HR = 1.765; 95% CI 1.536–2.029; *p* = 0.001). The increased risk of rehospitalization for HF at 36 months was still observed after multivariable adjustment (i.e., ≥80 years: HR = 1.265; 95% CI 1.049–1.524; *p* = 0.014; 60–<80 years: HR = 1.339; 95% CI 1.145–1.565; *p* = 0.001) and was specifically evident in patients with left ventricular ejection fraction ≥ 35% and in patients without evidence of CAD/single-vessel CAD. In contrast, the rates of AMI and coronary revascularization at 36 months did not differ significantly among different age groups. **Conclusions**: Advanced age is an independent predictor of rehospitalization for HF in patients undergoing CA, but not AMI and revascularization during long-term follow-up. This highlights the importance of optimizing diagnostic and therapeutic strategies for HF, particularly in older patients undergoing CA.

## 1. Introduction

Coronary artery disease (CAD) remains one of the leading causes of morbidity and mortality worldwide, with an increasing prevalence in older patients [1]. According to estimates from the United Nations, the global prevalence of people aged 65 or older will rise from 10% in 2022 to 16% in 2050 [2]. Therefore, an increase in the number of older patients undergoing coronary angiography (CA) can be expected. Due to age-related changes in organ function and morphology, as well as a higher burden of cardiac and non-cardiac comorbidities associated with age, the diagnosis and treatment of CAD in older people poses significant challenges for healthcare systems. Specifically, elderly patients have often been excluded from prior studies focusing on revascularization strategies. Because of this, data focusing on invasive strategies in this population remains limited [3].

The rupture of atherosclerotic plaques plays a major role in the pathogenesis of CAD, myocardial infarction (MI), and heart failure (HF) [4,5]. Because atherosclerotic plaques develop over an extended period of time, the burden of atherosclerosis increases with age. This is further aggravated by the increasing prevalence of important cardiovascular risk factors among older patients [6,7,8]. It has been shown that CAD mortality increases by a factor of 2.3 to 4.5 per decade of life while also being influenced by sex and the region of observation [9]. Previous studies have suggested that patients of different age groups possess significantly differing risk profiles, implying the need for specific and risk-profile-adjusted treatment strategies [8,10]. However, prior studies often either solely include patients with acute coronary syndrome (ACS) or explicitly exclude older patients [11,12,13,14]. Detailed data regarding age-related differences in the diagnosis and treatment of CAD is limited.

This study aims to investigate the relationship between age and both the presence and extent of CAD, as well as long-term outcomes in a large cohort of unselected patients undergoing invasive CA. Furthermore, the prognostic impact of age was stratified by the indication of CA.

## 2. Materials and Methods

### 2.1. Study Patients, Design, and Data Collection

For the present study, all consecutive patients undergoing CA at the University Medical Centre Mannheim (UMM), Germany, from January 2016 to August 2022 were included. Patients were identified using Operation and Procedure Classification (OPS) codes. The local electronic hospital information system (SAP^®^, Walldorf, Germany) was used to retrospectively document all relevant clinical data related to the index event, including symptoms and diagnosis on admission, prior medical history, and angiographic findings and interventions, as well as medication on discharge. Patients who underwent recurrent CA were only included once.

The UMM has a general emergency department to cater to emergency traumatic, surgical, neurological, and cardiovascular conditions. The cardiology department offers a 24/7 h catheterization laboratory, an electrophysiologic laboratory, a hybrid operating room, and telemetry units. Furthermore, the UMM is a part of an established network of clinics to which patients are sent who are in need of cardiac surgery, such as for coronary artery bypass grafts (CABGs).

### 2.2. Inclusion and Exclusion Criteria

For the present study, all consecutive patients ≥ 40 years of age undergoing invasive CA at our institution were included. CAs were performed by interventional cardiologists in accordance with current European guidelines [15]. CA operators were blinded to the final study analyses. For the present study, all source data of coronary angiographic examinations (imaging files) and reports were reassessed post hoc by two independent cardiologists. For the present study, risk stratification was performed comparing patients 40–<60 years, 60–<80 years, and ≥80 years. Patients < 40 years were excluded from the analysis due to their small number relative to the overall study cohort (171 out of 7691). No further exclusion criteria were applied.

### 2.3. Study Endpoints

The primary endpoint was rehospitalization for heart failure (HF) at 36 months. Secondary endpoints comprised the risk of acute myocardial infarction (AMI) and coronary revascularization at 36 months, as well as in-hospital all-cause mortality related to index hospitalization. All endpoints were defined using International Classification of Diseases (ICD) codes at the University Medical Centre Mannheim (UMM), Germany.

### 2.4. Statistical Methods

Quantitative data was presented as mean ± standard error of mean (SEM), median, and interquartile range (IQR), and ranges depending on the distribution of the data. They were compared using the Student’s t-test for normally distributed data or the Mann–Whitney U test for nonparametric data. Deviations from a Gaussian distribution were tested by the Kolmogorov–Smirnov test. Qualitative data was presented as absolute and relative frequencies and were compared using the Chi-square test or the Fisher’s exact test, where appropriate. Kaplan–Meier analyses investigating the risk of HF-related rehospitalization, coronary revascularization, and AMI were performed, including patients discharged alive. Univariable hazard ratios (HRs) were given, together with 95% confidence intervals.

The prognostic impact of age was thereafter investigated within multivariable Cox regression models. Multivariable Cox regression analyses were performed within the entire study cohort and also in pre-specified subgroups stratified by the presence of angina, (non-)ST segment elevation myocardial infarction ((N)STEMI), and decompensated HF at index hospitalization, multivessel CAD, and left ventricular ejection fraction (LVEF). Multivariable Cox regression analyses were visualized using forest plots. Multivariable Cox regression analyses within the entire study cohort were also performed, including age as a continuous variable (i.e., per 1-year increase).

The results of all statistical tests were considered significant for *p* ≤ 0.05. SPSS (Version 25 IBM, Armonk, NY, USA) was used for statistics.

## 3. Results

### 3.1. Study Population

From January 2016 to August 2022, 7520 patients undergoing CA at the catheterization unit of the University Medical Centre Mannheim were included in the present study. Patients’ mean age was 69 years (median: 70 years; IQR 60–79 years). Baseline characteristics and comorbidities on admission are shown in Table 1. The proportion of male patients (40–<60 years of age: 75.1% vs. 60–<80 years of age: 63.3% vs. ≥80 years of age: 55.8%; *p* = 0.001) as well as the rates of hyperlipidemia (44.1% vs. 33.7% vs. 29.1%; *p* = 0.001) decreased with age. In contrast, the rates of arterial hypertension increased in older patients (81.4% vs. 87.0% vs. 87.5%; *p* = 0.001). During index hospitalization, the highest rates of concomitant NSTEMI were found in patients aged ≥80 years, followed by patients aged 60–<80 and 40–<60 years (21.0% vs. 17.8% vs. 16.9%; *p* = 0.005). In contrast, unstable angina (21.9% vs. 25.5% vs. 32.1%; *p* = 0.001) and STEMI (7.9% vs. 10.0% vs. 18.3%; *p* = 0.001) were less frequently found in older patients.

Furthermore, the rates of moderately (i.e., 35–44%) (10.1% vs. 14.5% vs. 18.4%; *p* = 0.001) and severely reduced LVEF (i.e., <35%) (12.2% vs. 16.0% vs. 16.0%; *p* = 0.001) increased with age.

Angiographic findings, as well as clinical outcomes, are presented in Table 2. Patients aged ≥ 80 years exhibited the highest prevalence of CAD, followed by patients aged 60–<80 years and 40–<60 years (77.3% vs. 71.8% vs. 61.9%; *p* = 0.001), as well as the highest rate of two-vessel (22.4% vs. 20.9% vs. 19.1%; *p* = 0.001) and three-vessel disease (36.1% vs. 31.5% vs. 21.0%; *p* = 0.001).

**Figure 1:**
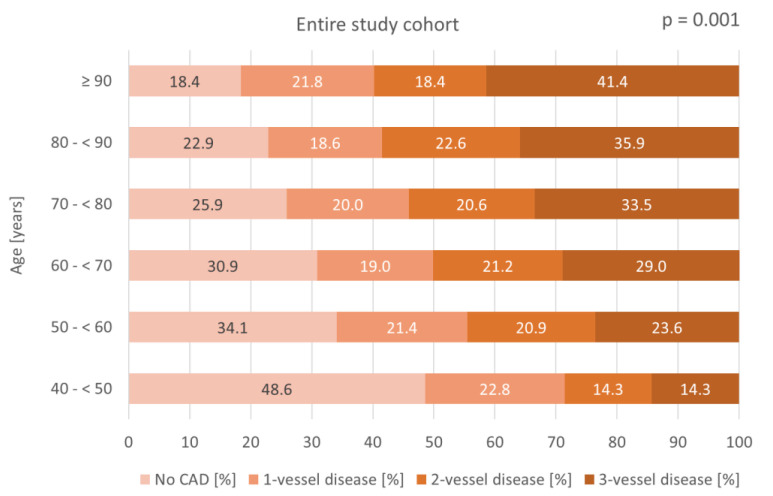
Bar chart displaying the distribution of CAD severity across age groups in 10-year increments within the entire study cohort.

Figure 1 shows significantly higher CAD prevalences in older patients within the entire study cohort when stratifying patient age into 10-year age increments: 51.4% in patients aged 40–<50 years compared to 81.6% in patients aged ≥ 90 years (*p* = 0.001). The prevalence of three-vessel disease rises from 14.3% to 41.4% when comparing the same age groups (*p* = 0.001).

**Figure 2:**
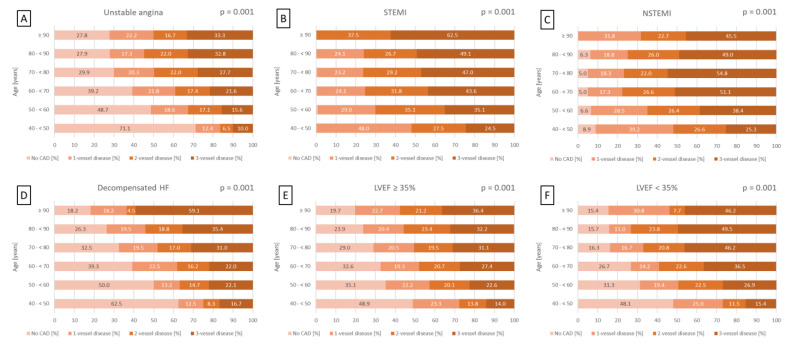
Bar charts displaying the distribution of CAD severity across age groups in 10-year increments within subgroups of patients presenting with unstable angina (**A**), STEMI (**B**), NSTEMI (**C**), decompensated HF during index hospitalization (**D**), LVEF ≥ 35% (**E**), and LVEF < 35% (**F**). HF, heart failure; LVEF, left ventricular ejection fraction; (N)STEMI, (non-)ST segment elevation myocardial infarction.

This trend was also seen in different patient subgroups. In patients with LVEF ≥ 35%, the rates of CAD and three-vessel disease rise from 51.1% to 80.3% and from 14.0% to 36.4%, respectively (*p* = 0.001) (Figure 2E). In patients with LVEF < 35%, CAD prevalence rises from 51.9% to 84.6%, and three-vessel CAD prevalence rises from 15.4% to 46.2% (*p* = 0.001) (Figure 2F). A significant age-related increase in the prevalence of CAD and three-vessel disease was also observed in patients presenting with unstable angina (*p* = 0.001), STEMI (*p* = 0.001), NSTEMI (*p* = 0.001), and decompensated HF at index hospitalization (*p* = 0.001) (Figure 2A–D).

However, no significant age-related differences in the need for PCI were observed when comparing patients aged 40–<60, 60–<80, and ≥80 years (43.1–43.7%; *p* = 0.908) (Table 2).

### 3.2. Prognostic Impact of Age in Patients Undergoing CA

**Figure 3:**
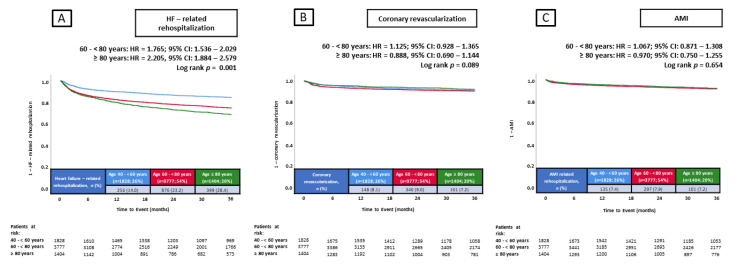
Prognostic impact of age in unselected patients on the risk of HF-related rehospitalization at 36 months (**A**), coronary revascularization at 36 months (**B**), and AMI at 36 months (**C**). AMI, acute myocardial infarction; CI, confidence interval; HF, heart failure; HR, hazard ratio.

The primary endpoint of this study, rehospitalization for HF at 36 months, occurred in 14.0% of patients 40–<60 years of age, in 23.2% of patients 60–<80 years of age, and in 28.4% of patients ≥ 80 years of age (Figure 3). Compared with patients 40–<60 years of age, patients 60–<80 years of age (HR = 1.765; 95% CI: 1.536–2.029; *p* = 0.001) and ≥80 years of age (HR = 2.205; 95% CI: 1.884–2.579; *p* = 0.001) had a higher risk of rehospitalization for HF at 36 months (Figure 3).

Additionally, the risk of in-hospital all-cause mortality increased with age (3.8% vs. 7.1% vs. 9.7%; *p* = 0.001) (Table 2). However, no significant differences in the risk of coronary revascularization (8.1% vs. 9.0% vs. 7.2%; *p* = 0.089) and AMI (7.4% vs. 7.9% vs 7.2%; *p* = 0.654) at 36 months were observed among the three age groups (Figure 3).

### 3.3. Multivariable Cox Regression Analyses

**Figure 4:**
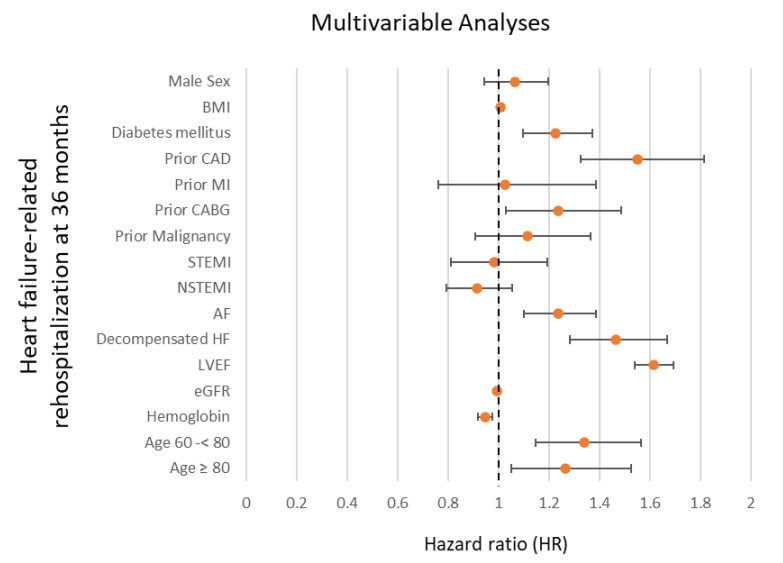
Forest plot displaying multivariable Cox regression analyses with regard to the risk of HF-related rehospitalization at 36 months within the entire study cohort. AF, atrial fibrillation; BMI, body mass index; CABG, coronary artery bypass grafting; CAD, coronary artery disease; eGFR, estimated glomerular filtration rate; HF, heart failure; LVEF, left ventricular ejection fraction; MI, myocardial infarction; (N)STEMI, (non-)ST segment elevation myocardial infarction.

After multivariable adjustment, advanced age was independently associated with a higher risk of rehospitalization for HF (i.e., 60–<80 years: HR = 1.339; 95% CI 1.145–1.565; *p* = 0.001; ≥80 years: HR = 1.265; 95% CI 1.049–1.524; *p* = 0.014) (Figure 4). Additionally, the presence of diabetes mellitus (HR = 1.226; 95% CI 1.096–1.372; *p* = 0.001), prior CAD (HR = 1.550; 95% CI 1.325–1.813; *p* = 0.001), prior CABG (HR = 1.236; 95% CI 1.029–1.485; *p* = 0.024), atrial fibrillation (HR = 1.235; 95% CI 1.101–1.385; *p* = 0.001), acute decompensated HF during index hospitalization (HR = 1.464; 95% CI 1.284–1.669; *p* = 0.001), and LVEF < 35% (HR = 1.614; 95% CI 1.538–1.694; *p* = 0.001) were associated with a higher risk of rehospitalization for HF at 36 months (Figure 4). However, a higher glomerular filtration rate (eGFR) (HR = 0.995; 95% CI 0.993–0.998; *p* = 0.001; per 1 mL/min increase) and higher hemoglobin levels (HR = 0.947; 95% CI 0.920–0.974; *p* = 0.001; per 1 g/dL increase) were associated with a lower risk of HF-related rehospitalization (Figure 4).

Even when included as a continuous variable, advanced age was still associated with a higher risk of HF-related rehospitalization at 36 months (HR = 1.010, 95% CI 1.004–1.015, *p* = 0.001, per 1-year increase).

**Figure 5:**
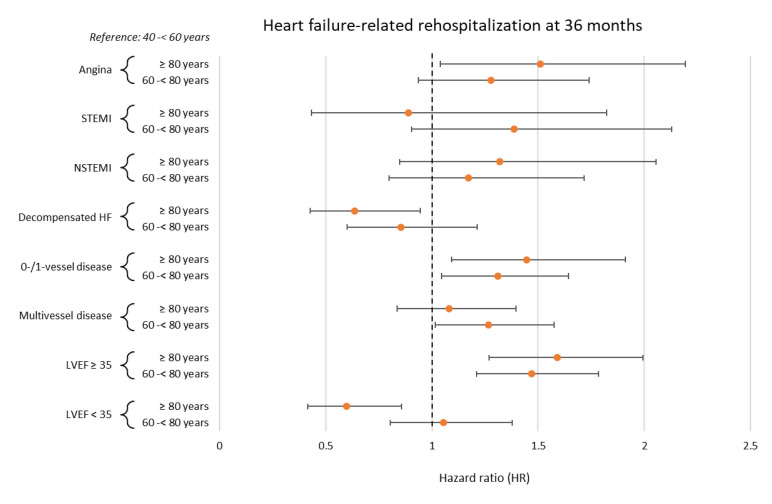
Forest plot displaying subgroup analyses investigating the prognostic impact of age with regard to the risk of HF-related rehospitalization at 36 months. HF, heart failure; LVEF, left ventricular ejection fraction; (N)STEMI, (non-)ST segment elevation myocardial infarction.

Within the following subgroup analyses, advanced age was associated with a significantly higher risk of HF-related rehospitalization at 36 months in patients with no evidence of CAD or single-vessel disease (60–<80 years: HR = 1.310, 95% CI 1.045–1.644; *p* = 0.019; ≥80 years: HR = 1.445, 95% CI 1.093–1.911; *p* = 0.010) and in patients with a LVEF ≥ 35% (60–<80 years: HR = 1.469, 95% CI 1.211–1.783; *p* = 0.001; ≥80 years: HR = 1.591; 95% CI 1.270–1.992; *p* = 0.001) (Figure 5).

## 4. Discussion

The aim of the present study was to investigate age-related differences in the presence and extent of CAD, as well as long-term outcomes in a large cohort of unselected patients undergoing invasive CA from 2016 to 2022. The main findings of our study can be summarized as follows: the prevalence of CAD, particularly multivessel CAD, increased with age and was highest in patients ≥80 years of age. Patients with ≥80 years of age had the highest risk of rehospitalization for HF at 36 months, as well as the highest risk of in-hospital all-cause mortality. This was especially evident in patients without evidence of CAD or single-vessel disease and in patients with LVEF ≥ 35%. However, the rates of AMI and coronary revascularization did not differ among different age groups during 36 months of follow-up.

The growth of atherosclerotic plaques, which is the main driving force in the development and progression of CAD, accelerates with increasing age [16]. Older patients typically present with a higher plaque volume and greater plaque calcification than younger patients [7,17,18]. While young patients undergoing CA typically present with higher rates of obesity and hyperlipidemia, the prevalences of diabetes mellitus and hypertension increase with advancing age [13,19,20,21]. A similar pattern in the distribution of the above-mentioned risk factors was observed in the present study. Accordingly, age represents an important prognostic factor in the development and progression of many cardiovascular diseases. This applies, in particular, to the development of HF, which, given recent and ongoing demographic changes, represents an ever-increasing global health problem [1]. In older patients, a reduction in cardiac reserve is often observed. This is caused by various age-related processes, such as decreased sensitivity to β-adrenergic stimulation, reduced compliance of blood vessels and cardiac tissue, and impaired energy metabolism of the myocardium [22,23].

The main causes of HF are arterial hypertension, CAD, and valvular heart disease, all of which are more prevalent in older patients [22]. Arterial hypertension leads to a higher afterload, concentric left ventricular hypertrophy and may ultimately lead to diastolic dysfunction and HF with preserved ejection fraction (HFpEF) [24,25]. Consistent with the increasing prevalence of hypertension with age, the incidence of HFpEF also increases with age, while HF with reduced ejection fraction (HFrEF) tends to occur more frequently in younger patients [26,27,28]. Aortic valve stenosis (AS) is another pathology that facilitates the development of HF, particularly when it is accompanied by mitral regurgitation (MR) or CAD. Because AS can contribute to the development of MR and both AS and CAD share common pathophysiological mechanisms, the coexistence of these three conditions is frequently observed [29,30]. The present study found that the percentage of concomitant valvular heart disease in patients undergoing CA substantially increases with age, affecting nearly one-third of patients ≥ 80 years of age. Due to a higher burden of the aforementioned comorbidities, as well as improved survival rates after AMI in older patients and a consequent higher number of older patients possibly developing HF in the years following a myocardial infarction, the overall prevalence and mortality of HF increase significantly with age [22,31,32,33].

Furthermore, older patients undergoing CA present more frequently with NSTEMI and multi-vessel disease, while younger patients are more likely to present with STEMI and single-vessel disease [13,14,19,20,21]. Advanced age is a well-known independent predictor of increased short-term and long-term mortality in patients undergoing CA [14,20,34,35]. However, no significant age-related differences with regard to the risk of reinfarction were observed in previous studies [34,35]. This is consistent with our findings. While we found no significant differences in the risk of reinfarction or revascularization between age groups, we identified a significant age-dependent difference in the risk of rehospitalization for HF in patients undergoing CA.

Prior studies examining the outcomes of patients with suspected CAD predominantly included patients experiencing acute coronary syndrome (ACS) or AMI [13,14,34,35]. Data was commonly provided for the rates of HF upon admission or the risk for the development of in-hospital HF, but not for the long-term risk of developing HF after AMI [20,34,36].

As mentioned above, CAD is one of the leading causes of the development of HF. Conversely, HF is also a relatively common comorbidity in patients admitted for CA [37]. Furthermore, HF has been identified as an independent predictor of increased mortality in ACS patients [20,37]. Steg et al. found that the presence of HF on admission has the highest impact on mortality in patients <55 years of age compared to older patients [37]. Nevertheless, older patients undergoing CA are significantly more likely to present with HF on admission or to develop symptoms of HF during index hospitalization [20,34,36,38]. Finally, it has been shown that older patients are also at higher risk of developing HF in the years after receiving CA [38,39,40]. This is in line with our findings that patients aged 60–<80 years or ≥80 years are at higher risk of developing HF compared to patients aged 40–<60 years.

However, an increasing burden of comorbidities, as well as age-related remodeling processes and reduced cardiac reserve, contribute to an increased risk of HF-related rehospitalization after CA in older patients. In our study, this effect was especially seen in patients with no evidence of CAD or single-vessel disease and in patients with an LVEF ≥ 35%. Advanced age has been associated with an increased risk of long-term mortality both in patients with HFrEF [41,42] and HFpEF [43,44]. It has also been reported that the prognosis of older patients with HFrEF is worse than that of older patients with HFpEF [45,46]. Knowing this, patients with a higher risk, i.e., patients with severely reduced LVEF and/or multivessel disease, may die before being hospitalized for HF, which may partly explain the low impact of age on the risk of HF-related rehospitalization in these subgroups in the present study.

In light of ongoing demographic changes, optimizing the diagnosis and treatment of CAD and HF, as well as of cardiac and non-cardiac comorbidities, is becoming increasingly important, especially in older patients. Considering all of the above-mentioned phenomena and trends, patients ≥ 60 years of age should be regularly monitored following CA, even in patients with no evidence of obstructive CAD or single-vessel CAD, as well as in patients with LVEF ≥ 35%.

### Study Limitations

The study has several limitations. Firstly, all data, including follow-up data, were sourced solely from hospital records. Additionally, patients’ prior medical histories were assessed using OPS codes, which might lead to lower documented event rates of pre-existing illnesses. A major limitation of this study was the unavailability of data regarding long-term all-cause mortality beyond index hospitalization—especially considering the risk interaction between rehospitalization and death. Moreover, the design of this study was retrospective and single-center. As a result, despite multivariable Cox regression analyses, results may be influenced by measured and unmeasured confounding factors, including undiagnosed comorbidities, medication adherence, or lifestyle differences between the age groups. Additionally, despite the consecutive inclusion of patients, biases may arise from the selection of patients undergoing CA, as those with more severe or symptomatic CAD may have been preferentially selected. Another limitation was the fact that all endpoints were assessed at our institution only. Because of this, the results may not be generalizable to broader populations in different countries outside of Germany.

## 5. Conclusions

Higher age represents an independent predictor of rehospitalization for HF at 36 months in patients undergoing CA, particularly among those without evidence of CAD or single-vessel disease, and an LVEF ≥ 35%. In contrast, age did not predict the risk of AMI and coronary revascularization during long-term follow-up.

## Figures and Tables

**Table 1:** Baseline characteristics.

	Age 40–<60 yrs (*n* = 1901)		Age 60–<80 yrs (*n* = 4064)		Age 80 yrs (*n* = 1555)		*p* Value
**Age**, median (IQR)	54	(49–57)	71	(66–76)	83	(82–86)	**0.001**
**Male sex**, n (%)	1427	(75.1)	2574	(63.3)	868	(55.8)	**0.001**
**Body mass index,** kg/m^2^, median (IQR)	28.1	(25.1–32.1)	27.5	(24.5–31.1)	26.0	(23.6–29.3)	**0.001**
**Cardiovascular risk factors,** n (%)							
Arterial hypertension	1548	(81.4)	3534	(87.0)	1361	(87.5)	**0.001**
Diabetes mellitus	431	(22.7)	1346	(33.1)	508	(32.7)	**0.001**
Hyperlipidemia	839	(44.1)	1369	(33.7)	453	(29.1)	**0.001**
**Prior medical history**, n (%)							
Congestive heart failure	86	(4.5)	381	(9.4)	203	(13.1)	**0.001**
Pacemaker	11	(0.6)	71	(1.7)	33	(2.1)	**0.001**
COPD	31	(1.6)	200	(4.9)	66	(4.2)	**0.001**
Chronic kidney disease	41	(2.2)	232	(5.7)	146	(9.4)	**0.001**
Liver cirrhosis	26	(1.4)	54	(1.3)	8	(0.5)	**0.026**
Malignancy	45	(2.4)	286	(7.0)	114	(7.3)	**0.001**
Stroke	5	(0.3)	31	(0.8)	23	(1.5)	**0.001**
**Comorbidities at index hospitalization**, n (%)							
Acute coronary syndrome							
Unstable angina	610	(32.1)	1037	(25.5)	340	(21.9)	**0.001**
STEMI	347	(18.3)	406	(10.0)	123	(7.9)	**0.001**
NSTEMI	321	(16.9)	723	(17.8)	326	(21.0)	**0.005**
Atrial fibrillation	226	(11.9)	1142	(28.1)	635	(40.8)	**0.001**
Atrial flutter	8	(0.4)	106	(2.6)	48	(3.1)	**0.001**
Acute decompensated heart failure	92	(4.8)	496	(12.2)	330	(21.2)	**0.001**
Cardiogenic shock	52	(2.7)	185	(4.6)	77	(5.0)	**0.001**
Atrioventricular block	10	(0.5)	102	(2.5)	81	(5.2)	**0.001**
Cardiopulmonary resuscitation	120	(6.3)	296	(7.3)	112	(7.2)	0.374
Out-of-hospital	93	(4.9)	201	(4.9)	74	(4.8)	0.959
In-hospital	27	(1.4)	95	(2.3)	38	(2.4)	**0.046**
Valvular heart disease	116	(6.1)	683	(16.8)	488	(31.4)	**0.001**
Stroke	57	(3.0)	168	(4.1)	61	(3.9)	0.098
**LVEF**, n (%)							
>55	961	(55.4)	1715	(47.7)	579	(41.5)	**0.001**
45–55%	386	(22.3)	784	(21.8)	337	(24.2)	
35–44%	175	(10.1)	522	(14.5)	256	(18.4)	
<35%	212	(12.2)	576	(16.0)	223	(16.0)	
Not documented	167		467		160		

COPD, chronic obstructive pulmonary disease; LVEF, left ventricular ejection fraction; IQR, interquartile range; (N)STEMI, (non-)ST segment elevation myocardial infarction. Level of significance *p* ≤ 0.05. Bold type indicates statistical significance.

**Table 2:** Procedural, laboratory, and follow-up data.

	Age 40–<60 yrs (*n* = 1901)		Age 60–<80 yrs (*n* = 4064)		Age ≥ 80 yrs (n = 1555)		*p* Value
**Coronary angiography,** n (%)							
No evidence of coronary artery disease	725	(38.1)	1145	(28.2)	353	(22.7)	**0.001**
1-vessel disease	414	(21.8)	792	(19.5)	293	(18.8)	
2-vessel disease	363	(19.1)	848	(20.9)	348	(22.4)	
3-vessel disease	399	(21.0)	1279	(31.5)	561	(36.1)	
Right coronary artery	754	(39.7)	2000	(49.2)	806	(51.8)	**0.001**
Left main trunc	113	(5.9)	487	(12.0)	243	(15.6)	**0.001**
Left anterior descending	865	(45.5)	2285	(56.2)	990	(63.7)	**0.001**
Left circumflex	636	(33.5)	1794	(44.1)	755	(48.6)	**0.001**
Ramus intermedius	153	(8.0)	485	(11.9)	207	(13.3)	**0.001**
CABG	22	(1.2)	132	(3.2)	73	(4.7)	**0.001**
Chronic total occlusion	119	(6.3)	374	(9.2)	121	(7.8)	**0.001**
**PCI**, n (%)	830	(43.7)	1753	(43.1)	679	(43.7)	0.908
Right coronary artery	345	(18.1)	681	(16.7)	243	(15.6)	0.136
Left main trunc	47	(2.5)	149	(3.7)	95	(6.1)	**0.001**
Left anterior descending	421	(22.1)	900	(22.1)	377	(24.2)	0.201
Left circumflex	265	(13.9)	586	(14.4)	228	(14.7)	0.819
Ramus intermedius	41	(2.2)	71	(1.7)	24	(1.5)	0.368
CABG	3	(0.2)	37	(0.9)	15	(1.0)	**0.003**
**Sent to CABG**, n (%)	76	(4.0)	223	(5.5)	39	(2.5)	**0.001**
**Procedural data**							
Number of stents, median (IQR)	2	(1–3)	2	(1–3)	2	(1–3)	0.599
Stent length, median (IQR)	44	(24–76)	44	(24–76)	44	(24–76)	0.648
Contrast, median (IQR)	109	(69–184)	116	(70–200)	121	(75–202)	**0.003**
**Baseline laboratory values**, median (IQR)							
Sodium, mmol/L	139	(138–141)	139	(138–141)	140	(138–141)	0.403
Potassium, mmol/L	3.92	(3.72–4.15)	3.96	(3.73–4.22)	3.95	(3.72–4.25)	**0.001**
Calcium, mmol/L	2.22	(2.14–2.31)	2.21	(2.13–2.30)	2.20	(2.11–2.29)	**0.001**
Creatinine, mg/dL	0.93	(0.80–1.08)	1.05	(0.87–1.36)	1.19	(0.94–1.64)	**0.001**
eGFR, mL/min/1.73 m^2^	81.5	(70.2–94.9)	66.3	(50.2–81.3)	53.0	(39.4–68.4)	**0.001**
Urea, mg/dL	31.2	(25.8–38.6)	39.4	(30.9–54.1)	48.9	(36.2–69.3)	**0.001**
Hemoglobin, g/dL	14.2	(12.9–15.2)	13.1	(11.5–14.3)	12.2	(10.7–13.4)	**0.001**
WBC count, × 10^9^/L	9.3	(7.4–11.6)	8.8	(7.1–11.2)	8.7	(6.9–11.1)	**0.001**
Platelet count, × 10^9^/L	245	(204–292)	234	(191–284)	220	(179–267)	**0.001**
HbA1c, %	5.7	(5.4–6.3)	5.9	(5.5–6.8)	5.9	(5.5–6.7)	**0.001**
LDL-cholesterol, mg/dL	122	(95–148)	100	(77–131)	90	(68–120)	**0.001**
HDL-cholesterol, mg/dL	40	(33–49)	43	(35–54)	46	(37–56)	**0.001**
Triglycerides, mg/dL	143	(102–208)	126	(94–173)	148	(111–197)	**0.001**
C-reactive protein, mg/L	16.6	(7.1–55.2)	28.6	(9.0–87.0)	36.2	(12.0–94.6)	**0.001**
Procalcitonin, µg/L	0.30	(0.09–1.34)	0.43	(0.15–1.93)	0.30	(0.11–1.19)	**0.001**
Albumin, g/L	35.8	(33.0–38.5)	34.1	(30.2–37.0)	32.5	(29.1–35.5)	**0.001**
INR	1.03	(0.99–1.09)	1.06	(1.01–1.15)	1.09	(1.02–1.21)	**0.001**
NT-pro BNP, pg/mL	699	(156–2359)	2188	(577–6075)	3859	(1580–9633)	**0.001**
Cardiac troponin I, µg/L	1.48	(0.16–12.02)	0.59	(0.10–4.51)	0.54	(0.09–3.04)	**0.001**
Creatine kinase, U/L	162	(96–396)	126	(78–271)	121	(75–245)	**0.001**
Creatine kinase MB, U/L	35	(22–80)	31	(21–64)	30	(20–53)	**0.001**
**Medication at discharge**, n (%)							
ACE inhibitor	980	(53.6)	1900	(50.3)	691	(49.2)	**0.024**
ARB	311	(17.0)	977	(25.9)	416	(29.6)	**0.001**
Beta-blocker	1203	(65.8)	2736	(72.4)	1047	(74.6)	**0.001**
Aldosterone antagonist	230	(12.6)	590	(15.6)	228	(16.2)	**0.004**
ARNI	27	(1.5)	39	(1.0)	10	(0.7)	0.104
SGLT2 inhibitor	96	(5.3)	200	(5.3)	45	(3.2)	**0.005**
Statin	1271	(69.5)	2902	(76.8)	1058	(75.4)	**0.001**
ASA	1234	(67.5)	2486	(65.8)	854	(60.8)	**0.001**
P2Y12 inhibitor	862	(47.2)	1807	(47.8)	687	(48.9)	0.604
OAC	210	(11.5)	1162	(30.8)	611	(43.5)	**0.001**
**Follow-up data**, median (IQR)							
Hospitalization time	6	(4–9)	7	(5–13).	9	(5–15)	**0.001**
ICU time	0	(0–0)	0	(0–0)	0	(0–0)	0.127
**All-cause mortality, in hospital**, n (%)	73	(3.8)	288	(7.1)	151	(9.7)	**0.001**
**Patients discharged alive**, n (%)	1828	(96.2)	3776	(92.9)	1404	(90.3)	
**Primary endpoint**, n (%)							
HF-related rehospitalization, at 36 months	256	(14.0)	876	(23.2)	399	(28.4)	**0.001**
**Secondary endpoints**, n (%)							
Coronary revascularization, at 36 months	148	(8.1)	340	(9.0)	101	(7.2)	0.098
Acute myocardial infarction, at 36 months	135	(7.4)	297	(7.9)	101	(7.2)	0.663

ACE, angiotensin-converting enzyme; ARB, angiotensin receptor blocker; ARNI, angiotensin receptor neprilysin inhibitor; ASA, acetylsalicylic acid; CABG, coronary artery bypass grafting; DOAC, directly acting oral anticoagulant; eGFR, estimated glomerular filtration rate; HbA1c, glycated hemoglobin; HDL, high-density lipoprotein; ICU, intensive care unit; IQR, interquartile range; LDL, low-density lipoprotein; NT-pro BNP, aminoterminal pro-B-type natriuretic peptide; PCI, percutaneous coronary intervention; SGLT2, sodium glucose linked transporter 2; WBC, white blood cell. Level of significance: *p* ≤ 0.05. Bold type indicates statistical significance.

## Data Availability

The datasets used and/or analyzed during the current study are available from the corresponding author upon reasonable request.
